# Postural Abnormalities in Children with Congenital Zika Syndrome-Related Neurological and Visual Impairment

**DOI:** 10.3390/v16121959

**Published:** 2024-12-20

**Authors:** Raíne Borba, Amanda Rodrigues, Camila V. Ventura, Cláudia Marques, Lucélia Nóbrega, Taciana Higino, Dalmir Santos, Juliana Sallum, Liana O. Ventura

**Affiliations:** 1 Paulista School Medicine, Federal University of São Paulo (Unifesp), Sao Paulo 04023-062, Brazil; raineborba@gmail.com; 2 Rehabilitation Center, Altino Ventura Foundation (FAV), Recife 50070-040, Brazil; 3 Department of Research, Altino Ventura Foundation (FAV), Recife 50070-040, Brazil; 4 Department of Ophthalmology, Altino Ventura Foundation (FAV), Recife 50070-040, Brazil; 5 Department of Ophthalmology, HOPE Eye Hospital, Recife 50070-040, Brazil; 6 Department of Ophthalmology, Federal University of São Paulo (Unifesp), Sao Paulo 04023-062, Brazil; juliana@pobox.com

**Keywords:** Congenital Zika syndrome, posture abnormalities, neurological and visual impairment

## Abstract

Deformities, body asymmetries, and muscle contractures are common consequences of atypical postural patterns in children with c ongenital Zika syndrome (CZS). This study aimed to evaluate the posture of children with CZS, considering their neurological and visual impairments. Ophthalmological assessment included binocular best-corrected visual acuity (BCVA) using Teller Acuity Cards II (TAC II) and an ocular motility evaluation. Postural alignment was measured using the PhysioCode Posture (PCP) app. Twenty-four children with CZS (12 [50.0%] female) were included, with a mean age of 6.8 ± 0.7 years (range, 4.0–7.0 years). The majority (79.2% [19/24]) had microcephaly at birth. Visual impairment was detected in 95.2% (20/21) of the children, with 85.0% (17/20) classified as moderate, severe, or blind. Shoulder asymmetry was observed in 95.8% (23/24) of participants, and 75.0% (18/24) presented abnormal postural alignment of the head and hips. Additionally, spinal deviations were found in 41.7% (10/24) of the children. All children with CZS exhibited asymmetries and improper postural patterns, which may result from a combination of neurological and visual impairments as well as environmental factors.

## 1. Introduction

The Zika virus (ZIKV) became known worldwide in 2015–2016 when it caused an outbreak in the Americas that started in the northeast of Brazil [1,2]. The ZIKV transmission can occur from mother to fetus during pregnancy, as well as through sexual contact, blood transfusion and blood products, and possibly through organ transplantation [3,4]. Children exposed to ZIKV in utero may present microcephaly, cerebral abnormalities, and ocular findings, among other birth defects, as well as functional disabilities secondary to c entral nervous system (CNS) damage, also known as congenital Zika syndrome (CZS) [5]. These congenital malformations observed in children with CZS are challenging for health professionals and caregivers as they have severe implications on the neurodevelopment as well as the quality of life of the affected children and their caregivers [5,6].

Children born with CZS display atypical motor development. They present changes in muscle tone, motor control, and posture [7]. These changes result from dysfunction of the CNS, which disrupts the development of a robust musculoskeletal system necessary for the child to achieve an upright posture and support their own body weight against gravity [8,9]. Arthrogryposis, hyperreflexia, balance deficits, and asymmetries in the hip, knee, ankle, wrist, and shoulder joints may also be present in this population and negatively contribute to motor development and lead to increasingly frequent immobility [10,11].

Studies have shown that children with CZS have significant motor impairments, which reduce their opportunities to develop social and environmental exploration skills [10,11,12]. Furthermore, the severity of the physical disability causes significant biomechanical misalignments in the posture [12]. The appearance of deformities, joint stiffness, asymmetries, muscle contractions and shortening, and chronic pain are consequences of these atypical postural patterns [10,11,12].

Overlapping with these neurological manifestations, children with CZS present visual impairment [13]. Studies show that regardless of the structural damage caused by the ZIKV to the retina and optic nerve, most children with CZS present moderate to severe visual impairment, also known as cerebral/cortical visual impairment (CVI) [14,15]. When present, visual impairment can affect the child’s ability to integrate visual information with the proprioceptive and vestibular systems that are essential for balance, spatial orientation, and environmental perception [16]. The implications of an affected visual system extend beyond vision alone, potentially encompassing a child’s ability to aim accurately and develop and maintain proper body alignment and balance skills, with significant repercussions on their posture [17].

Body posture is strongly influenced by muscular and sensory factors including the performance of the visual system [18]. This complex neuromuscular function plays a fundamental role in balance, motor activities, and social participation. It is reasonable to assume that children with CZS are at greater risk of developing specific postural alterations, related to their disabilities and disorders [19]. These can manifest as postural misalignments such as scoliosis, hyperlordosis, hyperkyphosis, asymmetries in weight distribution, and postural instability.

Understanding the postural abnormalities in children with neurosensorial conditions is crucial to improve clinical interventions, provide adequate support to children and their families, and ensure a better quality of life for all. Thus, the present study aimed to assess the posture of children with CZS by evaluating posture-related abnormalities in children with CZS, considering their neurological and visual impairments.

## 2. Materials and Methods

### 2.1. Design

This is an observational, quantitative, cross-sectional, exploratory study, employing a convenient sample of children with CZS.

### 2.2. Participants

Children were consecutively recruited from the multidisciplinary rehabilitation program of the Altino Ventura Foundation’s (FAV’s) Rehabilitation Center, in Recife, Brazil. The inclusion criteria included children with (1) positive test for ZIKV obtained either by real-time reverse transcription-polymerase chain reaction (rRT-PCR) or Immunoglobulin M antibody capture enzyme-linked immunosorbent assay (MAC-ELISA) performed on cerebrospinal fluid, and/or (2) clinical manifestations of CZS including microcephaly, subcortical calcifications, and ocular manifestations, and a positive report of arbovirus symptoms during pregnancy by mother. Children with positive serology for other congenital infections, such as toxoplasmosis, rubella, cytomegalovirus (CMV), syphilis, herpes, and human immunodeficiency virus (HIV) were excluded from the study.

### 2.3. Sociodemographic and Clinical Questionnaire

A standardized questionnaire was administered to parents/caregivers with the assistance of a trained technician due to limited literacy among participants in the sample. The data collected included sociodemographic and clinical characteristics of the children.

### 2.4. Measures

#### 2.4.1. Posture Assessment

**Figure 1:**
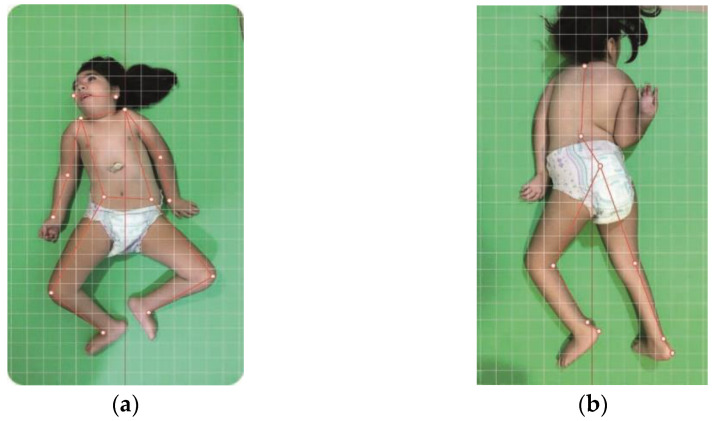
(**a**)—Anterior views of the first child with congenital Zika syndrome within a lying position. Marking of points for the evaluation of the head (earlobes), shoulder (acromion), elbows (epicondyle), wrists (radius bone), hip (iliac crests), knees (patella), ankle (malleolus). (**b**)—Posterior views of the first child within a lying position. Marking of points for the evaluation of the spine (C7 and T12 vertebrae) and knees (popliteal line) and ankles.

**Figure 2:**
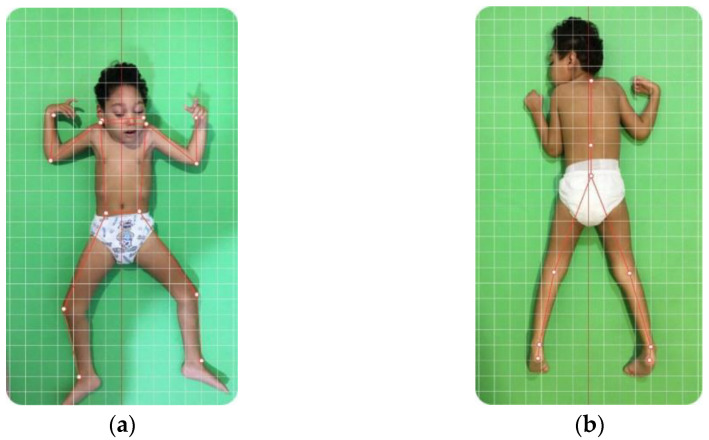
(**a**)—Anterior views of the second child with congenital Zika syndrome within a lying position. Marking of points for the evaluation of the head (earlobes), shoulder (acromion), elbows (epicondyle), wrists (radius bone), hip (iliac crests), knees (patella), ankle (malleolus). (**b**)—Posterior views of the first child within a lying position. Marking of points for the evaluation of the spine (C7 and T12 vertebrae) and knees (popliteal line) and ankles.

Posture alignment assessment was performed by using digital photographs captured and analyzed using the camera of an iPad (5 generations, version 14.4, model MPGT2LL/A) and the PhysioCode^®^ Posture(PCP) app [20], available for IOS and Android, with the position standardized at 100 × 20 cm from the floor and at 100 cm from the child. The children were placed on a 0.5 cm-thick mat on the floor, without accessories (e.g., watch, bracelets, hair clip, and glasses) and unclothed. Due to severe musculoskeletal impairment and/or neurological reflexes, some children were unable to remain in the prone and/or dorsal decubitus position in an aligned anatomic manner, and the position in which the child was allowed to remain without pain was respected (Figure 1 and Figure 2).

Data from the recorded images of each child assessed were analyzed using the PCP app algorithm, using measurements of and angular observations of the following variables: tilt of the head (earlobes), shoulder position (acromion), elbows (epicondyle), wrists (radius bone), hip (iliac crests), knees (patella and popliteal line), ankle (malleolus), and lateral deviation of the spine (C7 and T12 vertebrae) (Figure 1a,b and Figure 2a,b). The guidelines involved calibrating the image, marking the level of the joints and their specific characteristics mentioned above, generating the analysis report, and exporting it to Excel. The analysis report was generated automatically by the application, using the images collected, the demarcated points, and the registered reference (anatomical) values. In this way, the report provides information about the angulations found, whether they are within normal values, or the amount of deviation found (degrees).

#### 2.4.2. Visual Assessment

Children underwent a comprehensive ophthalmological examination performed by pediatric ophthalmologists using the same protocol described previously by Ventura et al. [12]. Binocular best-corrected visual acuity (BCVA) was assessed using Teller Acuity Cards II (TAC; Stereo Optical Co Inc., Chicago, IL, USA) and was recorded in cycles per cm (cy/cm). Visual acuity (VA) data were compared with age-related reference acuity norms and was categorized as very low vision (<1.6 cy/deg), low vision (1.6–9.6 cy/deg), near-normal vision (9.6–26.0 cy/deg), and normal vision (>26.0 cy/deg) [21]. Ocular motility examination included detecting strabismus and nystagmus. The angle of the strabismus was measured with the Krimsky test at near fixation (i.e., 33 cm), as children had significant visual impairment. Significant refractive errors included hyperopia of 2.00 diopters (D) or greater, or myopia or astigmatism of 1.00 D or greater [22].

### 2.5. Statistical Analysis

The characteristics studied were described by means of absolute and relative frequencies for qualitative variables, and by means of the mean and standard deviation for quantitative variables.

To assess the relationships between two qualitative variables, cross-contingency tables were constructed that present absolute values and percentages. In addition, Fisher’s exact test was used to verify the significance of the relationships. To assess the relationships between a qualitative and a quantitative variable, position measures, such as mean and median, and dispersion measures, such as standard deviation and minimum and maximum values for the quantitative variable, were calculated, considering each group of the qualitative variable. In addition, the Mann–Whitney test was used to verify the significance of the relationships.

In all cases, a significance level of 0.05 was considered statistically significant. Data processing and analysis were performed using Jamovi 2.3 software.

## 3. Results

Twenty-four children with CZS (12 [50.0%] female) were included in this study, with a mean age of 6.8 ± 0.7 years (range, 4.0–7.0 years). Microcephaly at birth was identified in 79.2% (19/24) of children, from which 73.7% (14/24) was classified as severe. All children (100.0%) had hypertonia, were using anticonvulsant medication, and 25.0% (6/24) presented developed hydrocephalus (Table 1). Most children (18 [75%]) were diagnosed with CZS due to positive serology for Zika virus. The remaining children (6 [25%]) had neuroimaging findings compatible with the syndrome, such as the presence of reduced brain volume, ventricular alterations, and calcifications (Table 2).

All children had postural dysfunctions. Twenty children (83.3%) presented with postural alterations in six or more corporal segments (tilt of the head, hip, shoulder position, lateral deviation of the spine, knee, and ankle).

Almost all children (23 [95.8%]) presented with shoulder position asymmetry: 10 (41.7%) had elevation of the right shoulder and 13 (54.2%) had elevation of the left shoulder. Most children (18 [75.0%]) presented alterations in the tilt of the head—seven children (29.2%) had right lateral flexion and eleven children (45.8%) had left lateral flexion. Alterations of the hip positioning was observed in most children (18 [75.0%]): eight children (33.4%) had elevation of the right hip and ten children (41.7%) had elevation of the left hip. Almost half of the children (10 [41.7%]) had lateral deviations of the spine—five (20.8%) with right convexity and five (20.8%) with left convexity, indicating the presence of thoracolumbar scoliosis on both sides. Nineteen children (79.2%) had knee deviation and 22 (91.7%) had ankle deviation (Table 3).

The mean knee deviation identified among the studied children was 48.7° (normal knee deviation: 0–8.9°), the mean head positioning deviation was 21.2° (normal head positioning deviation: 0–3.1°), the mean ankle deviation was 15.4° (normal ankle deviation: 0–7.4°), the mean hip asymmetry deviation was 14.5° (normal hip asymmetry deviation: 0–1.6°), the mean spine deviation was 7.5° (normal spine deviation: 0–5.0°), and the mean shoulder asymmetry deviation was 15.0° (normal shoulder asymmetry deviation: 0–1.6°).

Ophthalmological examination was performed in 21/24 children (87.5%). Visual impairment was present in 20/21 children (95.3%). Moderate visual impairment was detected in six [28.6%] children, severe visual impairment in six [28.6%] children, blindness in five [23.8%] children, and mild visual blindness in three (14.3%) children. Structural findings in the retina and/or optic disk were present in 8/21 (38.1%) children. Strabismus and nystagmus were seen in 15/21 (71.4%) and 4/21 (19.0%) of children, respectively. Significant refractive errors were present in all children (21 [100.0%]). Astigmatism was present in all children (21 [100.0%]) and hyperopia in 13 [61.9%] children. (Table 4). No correlation was found between the severity of visual impairment and postural deviations nor with greater angular deviations of body segments. (both *p*-values > 0.05) (Table 5 and Table 6).

Children with microcephaly did not present with more postural deviations nor with greater angular deviations of body segments compared to children without microcephaly (both *p*-values > 0.05). There was also no correlation between the severity of microcephaly and the presence of posture abnormalities (*p*-value > 0.05) (Table 5).

Children with microcephaly did not present with more postural deviations nor with greater angular deviations of body segments compared to children without microcephaly (both *p*-values >0.05). There was *n* ot a correlation between the severity of microcephaly and the presence of posture abnormalities (*p*-value >0.05) (Table 7).

The analysis of postural deviations in relation to the severity of microcephaly and visual impairment showed variations in frequency across the evaluated body segments. In cases of mild microcephaly, deviations were more frequent at mild and moderate levels of visual impairment, while, in more severe cases, higher prevalence was observed at severe and blindness levels. The head, shoulders, hips, knees, and ankles showed frequencies of postural changes ranging from 50.0% to 100.0%, with no statistically significant differences between groups (*p* > 0.05). Deviations in the spinal column occurred more frequently at moderate to severe levels of visual impairment in children with mild microcephaly (50.0 % to 100.0%) and at moderate and blindness levels in children with severe microcephaly (33.0% to 100.0%). Although the frequencies of postural changes varied according to body segment and the severity of microcephaly, no statistically significant correlation was observed with the severity of visual impairment (Table 8).

## 4. Discussion

Postural assessment in children with disabilities is complex and challenging. It requires a deep understanding of the multiple interactions between the musculoskeletal, sensory, and visual systems [23]. Although significant advances have been made in understanding postural alterations in children with disabilities, gaps in knowledge can be identified, especially when it comes to children with severe motor impairments that cannot sustain sitting or standing positions independently. The lack of specific assessment tools for children with neurological conditions and severe motor disabilities including CZS limits the ability to properly identify and quantify postural changes in this population.

The present study assessed the postural dysfunctions in children with CZS using the PhysioCode^®^ Posture app [20], a specific app that has been developed to measure postural deviations based on photographic evaluations; this app identified that all of the children presented with postural deviations in their body segments and more than 80.0% presented with alterations in more than six body segments.

The unalignment of the musculoskeletal system in children with CZS may have significant clinical and functional repercussions. In addition to functional implications such as mobility difficulties and challenges in performing daily activities, postural alterations can cause chronic pain and discomfort, resulting in negative psychosocial impacts [24,25]. Moreover, studies comparing postural alignment patterns in children with typical development with those with disabilities such as cerebral palsy revealed substantial differences that have significant impacts on their quality of life [24,25].

Pone et al. showed that these neurological complications in CZS create significant challenges for maintaining proper postures due to repercussions on the neuromuscular system [26]. Therefore, the postural findings in children with CZS observed here in could be a result of the structural damage caused by ZIKV to the brain that leads to hypertonia and consequently deviations of the body segments. In addition, vision plays a fundamental role in body development as it is a primary source of stimulus that enables direct interaction with the external environment [16]. The visual impairment identified in almost all children herein may have negatively contributed to the loss of spatial orientation, environmental perception, and children’s balance, favoring the persistence of atypical movement patterns and abnormalities of postures [27,28].

It was possible to identify that three out of every four children in our sample presented with hip positioning alterations. This finding corroborates with previous studies that demonstrated a high prevalence of hip alterations in children with CZS [28]. Additionally, almost half of the children (41.7%) had deviations in the spine, suggesting the presence of scoliosis. These alterations may indicate that more severe orthopedic impairments may develop over time, causing pain, deformity, and limitation of movement, affecting the child’s well-being [26,29].

Other significant body alterations were identified, such as foot deformities and knee joint alterations, previously reported by Matos et al. (2021) [30]. Most children presented deviations in knee and ankle joints. These findings highlight the extent of musculoskeletal complications, such as increased muscle tone, little experimentation with higher postures (standing posture and walking), and the prolonged maintenance of inadequate postures, contributing to the emergence of possibly surgical postural alterations.

Although no correlation was identified between severity of microcephaly and visual impairment with the postural alterations, it is known that posture alignment depends on the regulation and work of several systems at the same time. In children with multiple disabilities, broad manifestations, including neurological, visual, sensory, musculoskeletal, and proprioceptive manifestations, contribute negatively to postural alignment.

It is important to highlight the short- and long-term impacts that postural alterations bring to children. The psychosocial impacts, for example in play, school learning, nutrition, communication, feeding, sleep, and participation in physical activities, must be considered for individual well-being. Therefore, understanding the implications of postural alterations is fundamental to guide early and effective interventions that can prevent or correct these complications, to minimize the risk of musculoskeletal deformities causing pain and limiting social participation, to reduce the need for surgical procedures throughout life, and to improve the quality of life of children with CZS [31]. In this context, to minimize and even correct postural deformities early, the children included in this study were subject to specific and ongoing interventions, such as foot– ankle orthoses to prevent foot deformities, and physical therapy rehabilitation up to four times a month. The patients were also on the waiting list for corrective surgeries for orthopedic conditions which included correcting scoliosis, hip dysplasia, and dislocations.

The limitations of this study include the small sample size and the challenging assessment due to the difficulty in maintaining the children in standardized anatomical postures and without much external interference. The absence of a healthy control group is a limitation of our study too; however, we mitigated this limitation by thoroughly verifying Zika virus (ZIKV) diagnosis through laboratory testing and comprehensive clinical-epidemiological profiling. We also ruled out other conditions associated with similar symptoms by examining each child’s medical history and performing additional serological testing to exclude relevant differential diagnoses. It is noteworthy that the postural changes described herein may be postural compensations, due to the body rotations that these children present with and that are related to severe motor impairment. However, it is still important to be aware of this clinical picture so that the approach of health professionals can be more specific, and interventions can be made earlier and more assertive. Another limitation of the study was that most children presented with microcephaly and visual impairment in our sample. Therefore, statistical analyses to evaluate a relationship between the postural alterations and these variables were compromised. However, microcephaly, severe microcephaly, and visual impairment were observed in most cases, which imposes a limitation in the statistical analysis given the asymmetry between groups.

## 5. Conclusions

Children with CZS have significant body asymmetries and postural alterations that may lead to more complex long-term physical deformities and functional deficits. In addition, the postural misalignment herein observed may have a negative impact on the quality of life of children with CZS.

The implementation of specific assessments and early interventions to address the postural dysfunction in CZS are key points to improve the quality of life and well-being of this affected population.

## Figures and Tables

**Table 1:** Demographic and clinical data of children with congenital Zika syndrome (*n* = 24 children).

Variables	*n* (%)
Age in months (mean ± SD)	6.8 ± 0.7
Sex	
Male	12 (50.0)
Female	12 (50.0)
Microcephaly	
Yes	19 (79.2)
No	5 (20.8)
Severity of microcephaly	
≥3 SD and ≤2 SD	5 (26.3)
≤3 SD	14 (73.7)
Hypertonia	
Yes	24 (100)
No	0 (0)
Anticonvulsant medication	
Yes	24 (100)
No	0 (0)
Hydrocephalus	
Yes	6 (25.0)

SD, standard deviation.

**Table 2:** Diagnostic characterization of CZS: serology and neuroimaging findings (*n* = 24 children).

Patient	Zika Serology	Brain Imaging	Reduced Brain Volume	Lateral Ventricular Enlargement	Fourth Ventricular Enlargement	Third Ventricular Enlargement	Fissure Enlargement	Sulci Reduction	Lissencephaly	Agyria	Pachygyria	Calcifications
1	Yes	CT	No	Yes	No	No	No	No	No	No	No	Yes
2	Yes	CT	Yes	Yes	No	No	No	Yes	Yes	No	No	Yes
3	Yes	MRI	Yes	Yes	No	Yes	No	Yes	Yes	Yes	No	Yes
4	Yes	CT	Yes	Yes	No	No	No	No	No	No	No	Yes
5	Yes	CT	Yes	Yes	No	Yes	Yes	No	Yes	No	Yes	Yes
6	No	CT	No	Yes	No	Yes	No	No	No	No	No	Yes
7	Yes	CT	Yes	Yes	Yes	Yes	Yes	No	Yes	No	Yes	Yes
8	Yes	CT	Yes	Yes	No	Yes	Yes	No	Yes	No	Yes	Yes
9	Yes	CT	Yes	Yes	No	No	No	Yes	Yes	No	No	Yes
10	Yes	CT	Yes	Yes	No	Yes	No	Yes	Yes	No	No	Yes
11	Yes	MRI	Yes	Yes	Yes	Yes	No	Yes	Yes	No	Yes	Yes
12	Yes	CT	Yes	Yes	No	No	No	Yes	Yes	Yes	Yes	Yes
13	Yes	CT	Yes	Yes	Yes	Yes	No	No	Yes	No	No	Yes
14	Yes	CT	Yes	Yes	No	Yes	No	No	No	No	No	Yes
15	Yes	CT	Yes	Yes	Yes	Yes	Yes	Yes	Yes	No	Yes	Yes
16	Yes	CT	Yes	Yes	Yes	Yes	No	Yes	Yes	Yes	Yes	Yes
17	No	CT	Yes	Yes	Yes	Yes	No	No	No	No	No	Yes
18	Yes	CT	Yes	Yes	No	Yes	No	Yes	Yes	No	Yes	Yes
19	Yes	MRI	Yes	Yes	No	No	No	Yes	Yes	No	No	No
20	No	CT	No	Yes	No	No	No	No	No	No	No	Yes
21	No	CT	No	Yes	No	No	Yes	No	No	No	No	Yes
22	No	CT	No	Yes	Yes	Yes	Yes	No	No	No	No	Yes
23	Yes	MRI	Yes	Yes	Yes	Yes	No	Yes	Yes	No	Yes	Yes
24	No	MRI	Yes	Yes	Yes	Yes	No	Yes	Yes	No	Yes	Yes

CT = c omputed t omography; MRI = m agnetic resonance imaging.

**Table 3:** Posture abnormalities data of children with congenital Zika syndrome (*n* = 24 children).

Posture Abnormalities	*n* (%)
Head	
Left tilt	11 (45.8)
Right tilt	7 (29.2)
None	6 (25)
Shoulder	
Left elevation	13 (54.2)
Right elevation	10 (41.7)
None	1 (4.2)
Spine	
Left deviation	5 (20.8)
Right deviation	5 (20.8)
None	14 (58.3)
Hip	
Left elevation	10 (41.7)
Right elevation	8 (33.4)
None	6 (25)
Knee	
Varus	22 (84.6)
Valgus	10 (38.4)
None	2 (7.7)
Ankle	
Eversion	19 (73.1)
Inversion	14 (53.9)
None	0

**Table 4:** Ophthalmological findings data of children with congenital Zika syndrome (*n* = 21 children).

Ophthalmological Evaluation	*n* (%)
Visual impairment	
Normal	1 (4.8)
Mild	3 (14.3)
Moderate	6 (28.6)
Severe	6 (28.6)
Blindness	5 (23.8)
Structural findings	8 (38.1)
Retina	
Yes	7 (33.3)
No	1 (4.8)
Optic nerve	
Yes	6 (28.6)
No	2 (9.5)
Strabismus	
Yes	15 (71.4)
No	6 (28.6)
Nystagmus	
Yes	4 (19.0)
No	17 (81.0)
Refractive errors	
Myopia	8 (38.1)
Hyperopia	13 (61.9)
Astigmatism	21 (100)

**Table 5:** Postural deviation of body segments according to the visual impairment classification in children with congenital Zika syndrome (*n* = 21 children).

Body Segments	Visual Impairment Classification				
	Mild	Moderate	Severe	Blindness	*p*-Value *
Head	3/3 (100.0%)	5/6 (83.3%)	4/6 (66.7%)	3/5 (60.0%)	0.759
Shoulder	3/3 (100.0%)	5/6 (83.3%)	5/6 (83.3%)	5/5 (100.0%)	1
Hip	3/3 (100.0%)	5/6 (83.3%)	4/6 (66.7%)	3/5 (60.0%)	0.759
Right knee	3/3 (100.0%)	5/6 (83.3%)	4/6 (66.7%)	4/5 (80.0%)	0.910
Left knee	3/3 (100.0%)	6/6 (100.0%)	5/6 (83.3%)	4/5 (80.0%)	0.829
Spine	1/3 (33.3%)	4/6 (66.7%)	2/6 (33.3%)	2/5 (40.0%)	0.694
Left ankle	1/3 (33.3%)	6/6 (100.0%)	4/6 (66.7%)	4/5 (80.0%)	0.254
Right ankle	3/3 (100.0%)	5/6 (83.3%)	6/6 (100%)	5/5 (100.0%)	0.110

* Chi-Square test.

**Table 6:** The median of the angular deviation of body segments according to the visual impairment classification in children with congenital Zika syndrome (*n* = 21 children).

Body Segments	Visual Impairment Classification				
	Mild Median (Range)	Moderate Median (Range)	Severe Median (Range)	Blindness Median (Range)	*p*-Value *
Head	34.7 (14.3−36.7)	17.5 (1.0−176.9)	4.25 (0.3−19.1)	4.3 (0.8−30.8)	0.243
Shoulder	6.5 (1.9−18.4)	6.3 (1.1−173.3)	4.45 (1.6−17.4)	8.8 (4.0−12.2)	0.804
Hip	9.1 (7.1−17.7)	3.3 (0.3−175.2)	4.9 (0.0−10.5)	2.4 (0.2−8.9)	0.438
Right knee	40.9 (10.4−109.1)	39.25 (1.7−75.9)	49.65 (6.3−97.6)	25.6 (6.5−113.4)	0.971
Left knee	64.3 (60.5−66)	35.8 (21.2−117.1)	43.35 (6.5−135.4)	22.7 (7.1−107.5)	0.526
Spine	2.9 (0.2−12.9)	8.15 (0.1−15.4)	0.8 (0.7−17.7)	2.7 (0.5−37.2)	0.908
Left ankle	2.4 (2.1−13.6)	17.7 (15.1−21.2)	13.9 (0.7−54.2)	15.0 (2.5−25.9)	0.293
Right ankle	15.7 (8.6−19.1)	12.5 (2.8−30.8)	15.85 (7.5−28.8)	12.4 (9.8−47.1)	0.641

* Mann–Whitney test.

**Table 7:** The median of the angular deviation of body segments according to the extent of microcephaly in children with congenital Zika syndrome (*n* = 19 children).

Body Segments	Severity Microcephaly		*p*-Value *
	Less Severe Median (Range)	Severe Median (Range)	
Head	3.7 (0.3–17.9)	10.80 (0.8–176.9)	0.212
Shoulders	6.50 (1.7–10.5)	8.80 (1.6–173.3)	0.308
Hip	4.70 (1.7–9.1)	2.40 (0–175.2)	0.496
Right knee	10.40 (1.7–49.6)	28.90 (6.5–113.4)	0.145
Left knee	30.70 (6.5–64.3)	37.30 (7.1–107.5)	0.583
Spine	5.30 (0.2–15.4)	2.90 (0.5–37.2)	0.733
Left ankle	11.10 (0.7–21.2)	15.10 (1.9–54.2)	0.411
Right ankle	15.80 (9.2–19.9)	12.40 (2.8–47.1)	0.910

* Mann–Whitney test.

**Table 8:** Postural abnormalities/visual impairment between the microcephaly and non-microcephaly groups in children with congenital Zika syndrome (*n* = 19 children).

Microcephaly Classification	Postural Deviation	Visual Impairment Classification [*n*/total (%)]				*p*-Value
		Mild	Moderate	Severe	Blindness	
Mild	Head	1/1(100)	1/2(50)	1/2(50)	0/0(0)	1.00
Severe		1/1(100)	2/2(100)	2/3(66.7)	3/5(60)	1.00
Mild	Shoulder	1/1(100)	2/2(100)	2/2(100)	0/0(0)	0.545
Severe		1/1(100)	2/2(100)	2/3(66.7)	5/5(100)	1.00
Mild	Hip	1/1(100)	2/2(100)	2/2(100)	0/0(0)	1.00
Severe		1/1(100)	1/2(50)	1/3(33.3)	3/5(60)	1.00
Mild	Right knee	1/1(100)	1/2(50)	1/2(50)	0/0(0)	1.00
Severe		1/1(100)	2/2(100)	2/3(66.7)	4/5(80)	1.00
Mild	Left knee	1/1(100)	2/2(100)	1/2(50)	0/0(0)	1.00
Severe		1/1(100)	2/2(100)	3/3(100)	4/5(80)	1.00
Mild	Spine	0/1(0)	2/2(100)	1/2(50)	0/0(0)	0.600
Severe		0/1(0)	2/2(100)	1/3(33.3)	2/5(40.0)	0.610
Mild	Right ankle	0/1(0)	2/2(100)	1/2(50)	0/0(0)	0.600
Severe		0/1(0)	2/2(100)	2/3(66.7)	4/5(80)	0.515
Mild	Left ankle	1/1(100)	2/2(100)	2/2(100)	0/0(0)	1.00
Severe		1/1(100)	1/2(50)	3/3(100)	5/5(100)	0.273

## Data Availability

The data presented in this study are available on request from the corresponding author due to privacy or ethical restrictions.
